# A case of intravascular large B cell lymphoma presenting as nodular goiter

**DOI:** 10.1186/s13000-017-0656-x

**Published:** 2017-08-25

**Authors:** Bo Luo, Jia-mei Chen, Jie Liu, Wen-he Li, Yu-xiang Shi, Pan Zeng, Yong-hui Xie, Hong-feng Zhang

**Affiliations:** 1 Department of Pathology, The Central Hospital of Wuhan, Tongji Medical College, Huazhong University of Science and Technology, No. 26, Shengli Street, Wuhan City, Hubei Province 430014 People’s Republic of China; 2 Center of Oncology, Renmin Hospital of Wuhan University, No.99, Zhangzhidong Road, Wuhan City, Hubei Province 430060 People’s Republic of China

**Keywords:** Intravascular lymphoma, Large B-cell, Thyroid, Nodular goiter

## Abstract

**Background:**

Intravascular large B-cell lymphoma (IVLBCL) is a subtype of diffuse large B-cell lymphoma (DLBCL) that is rare and highly aggressive and that may progressively involve many organs. CNS (central nervous system), BM (bone marrow) and skin are the most common systems involved. To date, only 2 cases of IVLBCL involving the thyroid have been reported.

**Case presentation:**

Here, we report a case of IVLBCL involving the thyroid and accompanied by bilateral nodular goiter. In this case, a thyroid mass was identified in a physical examination of a 68-year-old male who initially presented with dyspnea accompanied by intermittent headache for approximately 1 month. Computed tomography scans revealed that the left lobar thyroid was occupied by a large, slightly lower density mass (5.8 × 4.7 × 8.4 cm). However, the patient had no hyperthyroidism or hoarseness. Levels of thyroid hormones and anti-thyroid autoantibodies in the serum were normal preoperatively. Thyroid mass resection was performed to establish a diagnosis and to relieve symptoms.

**Conclusions:**

Pathological results of the surgical specimen revealed that large atypical lymphoma cells filled the capillaries in the lesion area. Immunohistochemical staining revealed that the large-sized tumor cells were positive for CD20, PAX-5, MUM-1 and BCL-2, and were negative for CD3, CD5, CD43, CD10, CD23, CyclinD1, CD138, CD30, ALK, CD56, MPO, S-100, TTF-1, TG (thyroglobulin) and CT (calcitonin). The Ki-67 index was estimated to be approximately 85%. The patient was subsequently diagnosed as “Classical” IVLBCL non-germinal center B-cell type. The patient declined chemotherapy and died in the fifth month after operation.

## Background

Intravascular large B-cell lymphoma (IVLBCL) is a rare and highly aggressive subtype of extranodal DLBCL with an estimated annual incidence of fewer than 0.5 cases per 1,000,000 [1, 2]. IVLBCL was first described by Pfleger and Tappeiner [3] in 1959 and is characterized by the proliferation of malignant B-cells in small- and medium-size vessels. According to patients’ initial clinical presentation and bone marrow biopsy, the clinical phenotypes of IVLBCL are mainly classified into “Classical” IVLBCL and “Asian variant” IVLBCL [4–6]. “Classical” IVLBCL is characterized by CNS and/or cutaneous involvement. “Asian variant” IVLBCL is characterized by hemophagocytic syndrome, BM involvement, fever and hepatosplenomegaly.

IVLBCL can progressively involve any organ of the body [7–9]. In 2014, Fonkem et al. [2] retrospectively analyzed 740 cases of intravascular lymphoma (IVL) reported in the literature published worldwide between 1959 and 2011, among which 651 were IVLBCL. This retrospective research found that CNS, BM, spleen, skin, and lung were the most common systems involved, accounting for 60%, 11, 8, and 7% of cases, respectively. The involvement of other organs, such as the kidney, ovaries, uterus, and adrenal glands, has also been reported [10–12]. To our knowledge, only 2 cases of IVLBCL that involve the thyroid have been reported in the literature to date [13, 14]. One of these cases was a 68-year-old male who was admitted to the neurology department due to vertigo. A thyroid ultrasound revealed a left-sided nodule, and fine-needle aspiration cytology results revealed a papillary carcinoma in the left-sided nodule. Subsequently, the patient underwent a total thyroidectomy, and the histopathologic results revealed that IVLBCL presented as a dominant component [13]. The clinical-pathological details of the other case are unknown [14]. Here, we report a case of IVLBCL involving the thyroid. In this case, a 68-year-old male initially presented with dyspnea accompanied by intermittent headaches.

## Case presentation

### Clinical history

The 68-year-old male patient had a history of hypertension for approximately 20 years and chronic bronchitis for approximately 10 years. The patient was admitted to the respiratory department of our hospital because he had suffered from dyspnea and intermittent headaches for approximately 1 month. Physical examination revealed a 7 × 6 cm non-tender mass in the neck. No signs of hyperthyroidism or hoarseness existed. Neurological examination revealed no positive signs. Blood pressure (110/70 mmHg) was within the normal range. There was no family history of thyroid disease.

On admission, laboratory examination revealed the following: erythrocytes 3.94 (4.3–5.8 × 10^12^/L), hemoglobin content 124 (130–175 g/L), serum lactate dehydrogenase (LDH) 480.0 (15–240 U/L), hydroxybutyrate dehydrogenase 311.0 (50–220 U/L), total bilirubin 30.1 (5.1–20 μmol/L), direct bilirubin 11.6 (0.1–10 μmol/L) and indirect bilirubin 18.1 (3–15 μmol/L). All numbers in parentheses mentioned above indicate the reference range. Thyroid function tests revealed that serum calcitonin, thyroid hormones, thyroid stimulating hormone, and anti-thyroid autoantibodies were normal.

**Fig. 1:**
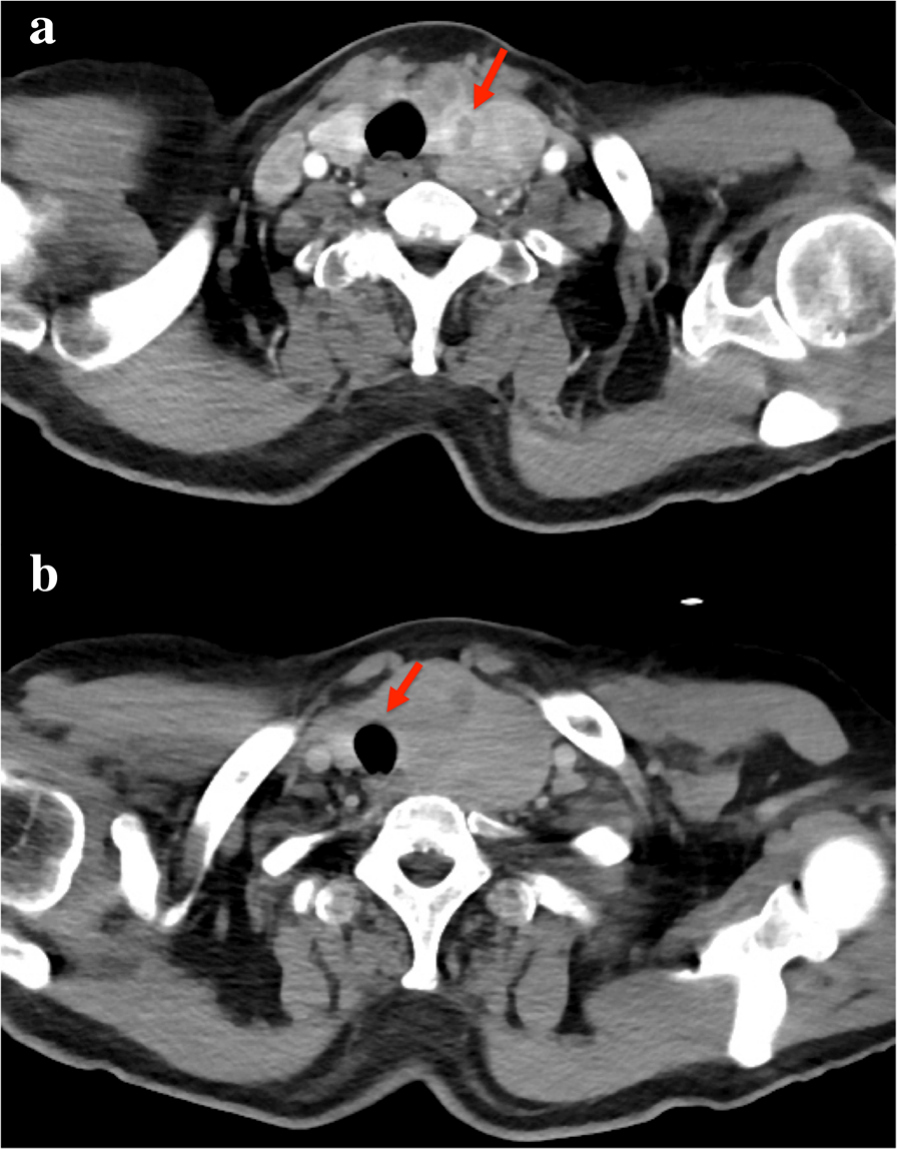
Computed tomography scan of the head and neck. a The left lobar thyroid is enlarged and is occupied by a large slightly lower density mass (5.8 × 4.7 × 8.4 cm). The mass expanded down to the chest entrance level. The main manifestations are scattered calcified lesions, flaky necrosis areas of low density, and inhomogeneous enhancement. b The trachea is pushed to the right by the remarkably enlarged thyroid

A CT (computed tomography) scan of the chest demonstrated bronchitis and bilateral bullae of lung. A CT scan of the head and neck revealed the following: 1) A slightly higher density nodule (1.5 cm) in the cerebellum caused a slight space-occupying effect; 2) Thyroid neoplasm invaded the anterior superior mediastinum. The left lobar thyroid was enlarged and occupied by a large, slightly lower density mass 5.8 × 4.7 × 8.4 cm in size. The mass protruded down to the chest entrance level, and the main manifestations were scattered calcified lesions, flaky necrosis areas of low density, and inhomogeneous enhancement (Fig. 1a). In the right lobar thyroid, there was a slightly higher density of round nodules (1.3 × 1.2 cm) without calcification or significant enhancement. The trachea was pushed by the giant mass to the right side and became narrow (Fig. 1b). There were no abnormal visible lymph nodes in either side of the neck. Thyroid color Doppler ultrasound revealed a giant hypoechoic mass (7.1 × 4.7 × 2.4 cm) with clear separation and heterogeneous echotexture in the left lobar thyroid and a hybrid echo-mass (1.3 × 1.2 × 1.0 cm) in the right side. CDFI exhibited an obvious blood flow signal in the lower density mass.

The patient was transferred to the thyroid breast surgery department. Thyroid mass resection was performed to establish a diagnosis and to relieve the symptoms caused by tumor compression. During the operation, the markedly enlarged thyroid was easily observed, and the trachea was pushed to the right. Multiple masses of various sizes were noted in the bilateral thyroid. The largest of these masses (10.0 × 6.0 × 5.0 cm) was noted in the left lobar thyroid, and 2/5 of the mass was behind the sternum with an intact capsule. In addition, a small (1.8 × 1.2 × 1.0 cm) mass with a distinct border was located in the middle of the right lobar thyroid. These masses were completely removed and sent to the pathology department for frozen section examination. Frozen section histological results revealed nodular goiter of bilateral and focal atypical hyperplasia in the left lobe. The patient then underwent a total thyroidectomy with central neck area lymph node dissection.

### Gross features

The entire thyroid had a complete packing membrane and a dark red appearance. The cutting appearance of the thyroid revealed that the giant mass (8.1 × 5.0 × 4.5 cm) in the left lobe was solid with medium hard features and an uneven texture. In addition, the 1.8 × 1.2 × 1.0 cm mass in the right lobe was gelatinous, soft, and uniform in texture. Both masses had a complete capsule. No visible enlargement of the lymph nodes was detected in the central neck area. Twenty paraffin blocks were cut from the total thyroidectomy specimen, 15 of which were cut from the left lobe and 5 from the right. However, only 3 paraffin blocks in the left lobe contained tumor cells as demonstrated by subsequent microscopic examination.

### Histological features

**Fig. 2:**
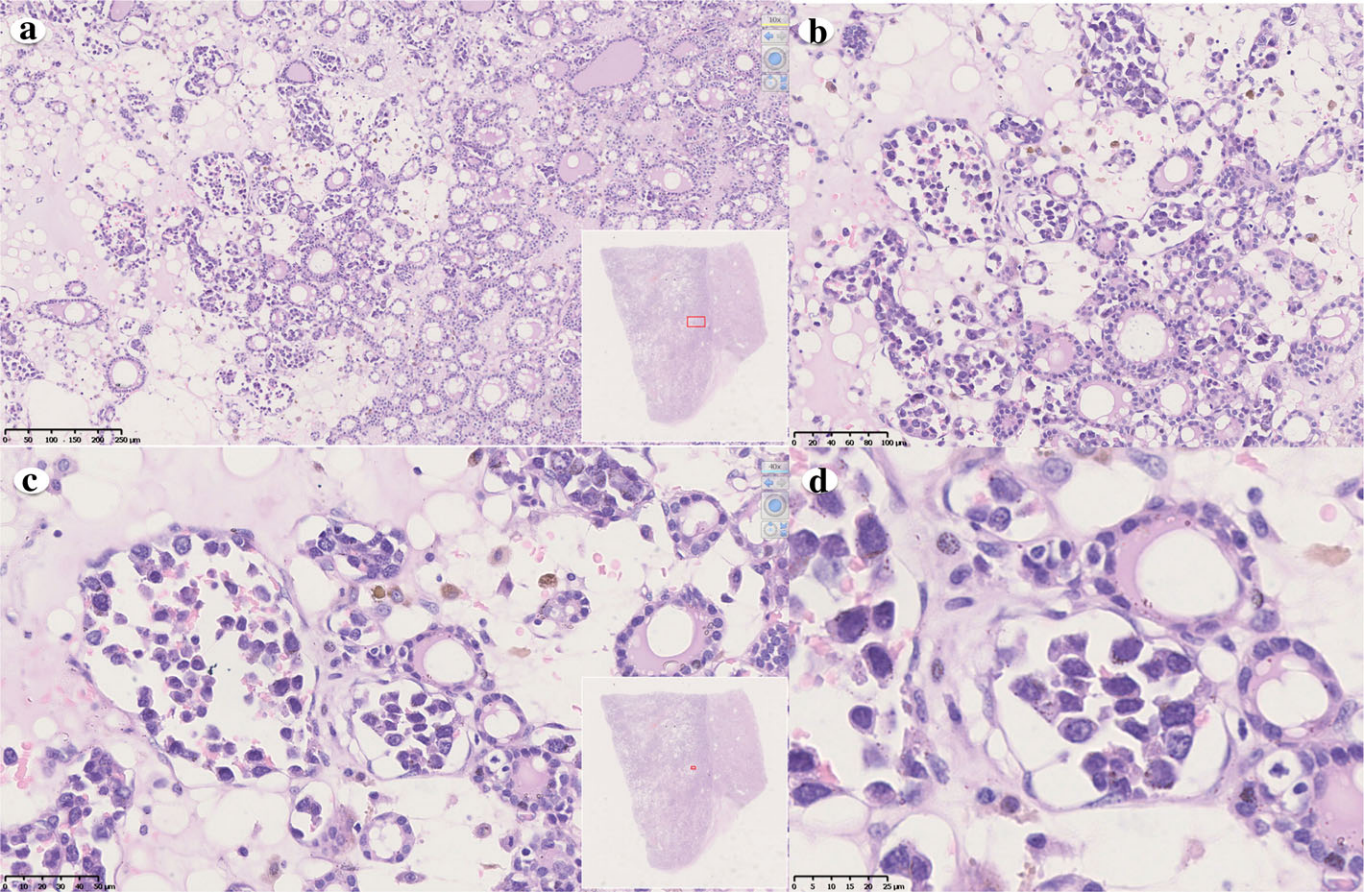
Histopathological features of thyroid intravascular large B-cell lymphoma. a Tumor cells are located in the interstitial of thyroid follicular without forming a clear boundary line (original magnification ×100). b, c Capillaries in the lesion area are filled by large atypical cells exhibiting an expansion appearance (original magnification ×200 and ×400 respectively). d Large-sized tumor cells with minimal cytoplasm, a thick nuclear membrane, irregular nuclear contours, prominent nucleoli and coarse to somewhat dispersed chromatin are noted (original magnification ×400)

All tumor cells were located in the interstitium of thyroid follicular cells without forming a clear boundary line, which appeared as an invasion of lymphocytes under low magnification (Fig. 2a). Thus, it might have been easy to miss the tumor under low magnification if pathologists had not been informed of the gross features. However, these tumor cells could be distinguished from mature small lymphocytes under lower magnification mainly based on their intravascular growth pattern, atypical nuclei and larger size. Capillaries in the lesion area filled by large atypical lymphoma cells exhibited an expanded appearance (Fig. 2b-c), which was quite different from normal capillary appearance. Under higher magnification, large-sized tumor cells exhibit minimal cytoplasm, a thick nuclear membrane, irregular nuclear contours, and prominent nucleoli and coarse to somewhat dispersed chromatin (Fig. 2c-d).

### IHC

**Fig. 3:**
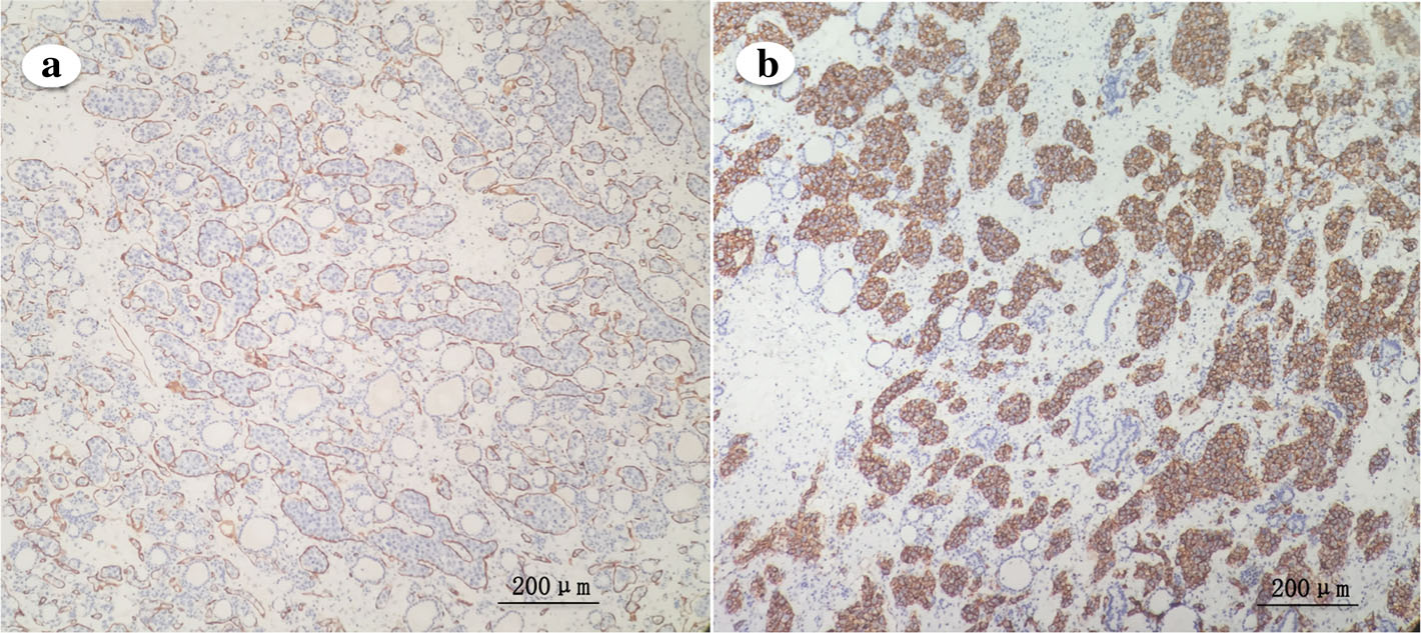
Immunohistochemistry staining of thyroid intravascular large B-cell lymphoma. a CD20-positive staining confirms that the large atypical lymphoma originates from B-cells (original magnification ×100). b CD34 immunohistochemistry staining highlights the intravascular growth pattern of tumor cells (original magnification ×100)

Immunohistochemical staining revealed that the large-sized tumor cells were positive for CD20, PAX-5, MUM-1 and BCL-2, and were negative for CD3, CD5, CD43, CD10, CD23, CyclinD1, CD138, CD30, ALK, CD56, MPO, S-100, TTF-1, thyroglobulin and calcitonin. The Ki-67 index was estimated to be approximately 85%. The intravascular growth pattern of the tumor cells was highlighted by CD34 staining of the endothelial cells (Fig. 3). CD20 and CD34 staining revealed that the tumor cells were not involved in the right lobe of the thyroid or the central neck area lymph nodes.

### Treatments and outcome

Based on the clinical history and histopathological and immunohistochemistry results, the patient was diagnosed as “Classical” IVLBCL non-germinal center B-cell type. The patient was recommended to receive the BCL-6/BCL-2 and MYC gene re-arrangement test and 6 cycles of Rituximab plus cyclophosphamide, doxorubicin, vincristine and prednisolone (R-CHOP) immunochemotherapy. However, the patient declined chemotherapy and died in the fifth month after operation.

## Discussion

IVLBCL is a rare malignant tumor that progresses rapidly and that may involve any organ, and IVLBCL involving the thyroid is particularly rare [2]. Depending on the organ involved, the clinical presentations of IVLBCL are non-specific and unpredictable, and may include neurological signs and symptoms, cutaneous lesions, fever, hepatosplenomegaly and cytopenia [5, 7, 15]. Given the high variance of clinical presentation, most patients undergo many investigations before the diagnosis is established. Occasionally, patients cannot be diagnosed or treated promptly as a result of the lack of sensitive diagnostic methods [5].

The patient in this case suffered from dyspnea and intermittent headaches for approximately 1 month. A giant non-tender mass in the neck was accidentally discovered upon physical examination. A CT scan demonstrated that the remarkably enlarged left lobar thyroid was occupied by a giant mass that suppressed the trachea. Thyroid function tests were normal preoperatively, whereas frozen section histological results revealed a nodular goiter with bilateral and focal atypical hyperplasia in the left lobe. Taking the increase in serum LDH and the occupied lesion in the cerebellum into consideration, we examined the tissue sections carefully. Eventually, we found lesion areas in three of the total 20 paraffin blocks. Thus, it is important to be aware of this entity and to examine the small vessels in biopsies as carefully as possible. In addition, the 18F–FDG PET/CT scan was demonstrated to have diagnostic value in IVLBCL [16, 17].

Histopathological differential diagnosis of IVLBCL mainly includes intravascular anastomotic large T-cell lymphoma [18], intravascular NK/T cell lymphoma [19], the other intravascular lymphoma with B-cell immunophenotype, such as exudative lymphoma, benign atypical intravascular CD30-positive T cell hyperplasia, and intravascular lymphatic tissue cell proliferation. Histopathological and immunohistochemistry staining results of this case were mostly typical, so no differential diagnosis was required. Regarding the clinical phenotype of the patient in our case, we might prefer the diagnosis of “Classical” IVLBCL to “Asian variant” IVLBCL, given that the patient did not experience fever, hepatosplenomegaly, hypoproteinemia or thrombopenia. The patient also had cerebellum involvement at the time of diagnosis according to medical history and CT results. Moreover, bone marrow biopsy is useful in making the correct classification of the clinical phenotype [20].

Due to the low morbidity of IVLBCL, prognostic factors of IVLBCL remain uncertain [21]. One study has suggested that a younger age (less than 70 years old), non-CNS site of initial diagnosis, LDH < 700 U/L and rituximab treatment were favorable prognostic factors [2]. By contrast, patients with hemophagocytic syndrome or CNS disease have a poorer prognosis [2, 22]. Other studies have reported that patients who had de novo CD5+ might present with aggressive disease, which signifies a very poor prognosis [23, 24]. In addition, some other potential prognostic factors might be involved, such as general physical state and extra-vascular involvement. Although IVLBCL is known for its rapid progression, some studies have suggested that long-term remission in patients with IVLBCL would be observed if they were diagnosed early and treated with conventional R-CHOP in time [15, 25, 26].

## Conclusions

In summary, we have presented the clinical features and natural course of primary thyroid IVLBCL. The patient in this case was diagnosed late with thyroid and cerebellum involvement. This case report suggests that clinicians and pathologists should be aware of the existence of thyroid IVLBCL. In addition, early diagnosis of IVLBCL through biopsy and treatment of patients with R-CHOP may be helpful to improve patients’ survival.

## Abbreviations

ALK: Anaplastic lymphoma kinase
BM: Bone marrow
CD: Cluster of differentiation
CDFI: Color Doppler flow imaging
CNS: Central nervous system
CT: Computed tomography
DLBCL: Diffuse large B-cell lymphoma
IVL: Intravascular lymphoma
IVLBCL: Intravascular large B-cell lymphoma
LDH: Serum lactate dehydrogenase
MPO: Myeloperoxidase
MUM-1: Multiple myeloma oncogene 1
PAX-5: Paired box protein 5
R-CHOP: Rituximab plus cyclophosphamide, doxorubicin, vincristine, and prednisolone
S-100: S-100 protein
TTF-1: Thyroid transcription factor 1
